# The efficacy of platelet-rich fibrin as a scaffold in regenerative endodontic treatment: a retrospective controlled cohort study

**DOI:** 10.1186/s12903-018-0598-z

**Published:** 2018-08-13

**Authors:** Hongbing Lv, Yuemin Chen, Zhiyu Cai, Lishan Lei, Ming Zhang, Ronghui Zhou, Xiaojing Huang

**Affiliations:** 1 School and Hospital of Stomatology, Fujian Medical University, Fuzhou, 350002 China; 2 Department of Stomatology, Fujian Medical University Union Hospital, Fuzhou, 350001 China

**Keywords:** Apical periodontitis, Human immature permanent tooth, Blood clot, Platelet-rich fibrin, Regenerative endodontic treatment

## Abstract

**Background:**

Blood Clot (BC) or platelet concentrates have been used as scaffold in regenerative endodontic treatment (RET). The aim of this retrospective study was to compare the performance of platelet-rich fibrin (PRF) with BC in inducing root development and periapical lesion healing after tooth revascularization.

**Methods:**

Five patients receiving RET using PRF as a scaffold were matched 1:1 to a previous cohort of 5 patients who underwent tooth revascularization by provoking periapical bleeding. Clinical signs and symptoms were examined at follow-ups. Periapical lesion healing and root development were monitored radiographically. The resolution of clinical signs and symptoms as well as periapical radiolucency was observed in all patients (100%).

**Results:**

Root elongation, dentinal wall thickening and apex closure were found in most cases (80% in both groups). There was no significant difference between the groups in terms of clinical sign resolution, root development and periapical healing.

**Conclusions:**

Within the limits of this study, PRF achieved comparable outcomes to BC in terms of clinical sign and symptom resolution, periapical lesion healing and continued root development in RET.

## Background

Traditionally, immature permanent teeth with necrotic pulp have been treated by apexification. In 2001, Iwaya et al. first reported a case involving the revascularization of an immature tooth with apical periodontitis [1]. Since then, a paradigm shift has occurred regarding the treatment of immature permanent teeth with pulp necrosis or apical periodontitis. In the past decade, several reports have described regenerative endodontic treatment (RET) [2–4]. Most of these studies were case reports or case series and presented successful results including the resolution of periapical lesions, continued root development, and even the recovery of tooth sensibility [5]. This accumulating evidence has contributed to the development of the current recommendations of the American Association of Endodontists for RET [6]. However, various protocols have been proposed, which differ regarding the concentrations of sodium hypochlorite (NaOCl) that are used for irrigation [7], the antibiotic regimens that are used for disinfection [5, 8], and the scaffold types that are used for tissue regeneration [9, 10]. Clearly, more evidence is needed to develop future treatment recommendations.

RET is based on the concept of tissue engineering [11], which requires the eradication of pathogens, the preservation of stem cells, and the presence of scaffolds and signal molecules [12]. To create a favourable microenvironment for stem cells to migrate, proliferate and differentiate, an ideal scaffold should facilitate spatial orientation and signal molecule release by cells. In most cases of tooth revascularization/revitalization, an endodontic explorer or file is introduced into the root canal and passes through the apical foramen to provoke bleeding from the periapical tissue into the canal to form a blood clot (BC) below the cemental enamel junction (CEJ) [13]. In general, this technique is effective at forming a BC scaffold. However, this procedure has several disadvantages. Firstly, the manipulation used to induce periapical bleeding is technique-sensitive. Clinically, it is difficult to control the speed and volume of bleeding to achieve the desired level. Too little bleeding would be insufficient to provide the necessary scaffold, whereas too much bleeding might overfill the pulp cavity and the open access, thus contacting the surrounding tooth crown and leading to recontamination of the disinfected root canal system. There are also clinical situations in which it is difficult to induce periapical bleeding [14]. Secondly, even when a BC is formed, such clots are far from ideal according to scaffold criteria. Lastly but most importantly, this procedure carries the risk of injury to the inferior alveolar nerve (IAN) or mental nerve when treating mandibular premolars. Studies have confirmed the proximity of premolar apices to the IAN and mental foramina [15–17]. It has been documented that iatrogenic mental nerve paresthesia can be caused by an overfill of Gutta-Percha or by mechanical instrumentation beyond the root apex [18, 19]. In addition, the presence of apical periodontitis or a radicular lesion might further erode the bone that protects the IAN. Therefore, it is imperative to find scaffold alternatives to a BC in RET.

Previous studies have shown the potential of using platelet concentrates as scaffolding in tissue regeneration. Platelet concentrates are autologous, reasonably easy to prepare in a dental setting, and comprise high concentrations of growth factors including transforming growth factor-beta (TGF-beta), vascular endothelial growth factor (VEGF), and platelet-derived growth factor (PDGF) [20]. In vitro studies have documented the effects of these signalling molecules on cell migration, proliferation, differentiation and matrix synthesis [21]. In recent years, platelet concentrates have been successfully applied as scaffolding in tooth revascularization/revitalization. Platelet-rich plasma (PRP) is a first-generation platelet concentrate [22]. Case reports [3, 23] as well as randomized controlled clinical studies [14, 24] have demonstrated the reliability of PRP in improving periapical healing, apical closure and dentinal wall thickening. Platelet-rich fibrin (PRF), a second-generation platelet concentrate, has many advantages over PRP. Firstly, the preparation of PRF does not require the addition of exogenous agents, such as thrombin. Secondly, PRF forms an organized fibrin network in which platelets and leukocytes are trapped. These entrapped cells serve as a reservoir of various growth factors for long-term release. Important circulating immune cells and various cytokines in PRF clots also act against infection. In addition, the mechanical properties of PRF might facilitate the condensation of overlying MTA. Thus, it is rational to expect PRF to be an optimal bioscaffold for tooth revascularization/revitalization.

However, apart from a few case reports describing the use of PRF as a scaffold [25–30], only one clinical trial [31] compared the efficacy of root development and periapical radiolucency resolution after tooth revascularization/revitalization with PRF and with other scaffolds. More clinical studies are required to evaluate the performance of PRF in RET. In order to test if PRF could enhance root development and periapical lesion healing more than a BC, we conducted a controlled cohort study.

## Methods

### Study population and design

A retrospective, serial case-control study design was used in this study. Patients having nonvital, immature incisors or premolars with radiographic evidence of periapical lesions were included. From January 2014 to December 2014, five cases of tooth revascularization with PRF application were performed by an experienced endodontist at the School and Hospital of Stomatology, Fujian Medical University. These patients represent an initial series of tooth revascularization cases using PRF as a scaffold that was conducted in our hospital in continuum without selection bias.

From January 2012 and December 2013, 11 conventional tooth revascularizations were performed by the same endodontist at the School and Hospital of Stomatology, Fujian Medical University. From this cohort, we selected 5 patients to serve as a control group (the remaining 6 cases were excluded for the following reasons: three cases involved pulp necrosis without periapical radiolucency; one patient underwent tooth extraction 10 months after RET for orthodontic reasons; in one case, tooth development was at stage 9; and contact was lost with the remaining patient after RET). These 5 patients were specifically matched in a 1:1 ratio to index cases of tooth revascularization using PRF with respect to patient age, gender, aetiology, pulp/periapical conditions, foramina development (All teeth were at stage 8 according to Nolla’s scoring system [32]) and tooth position. No consideration or analysis of operative parameters and outcomes was made until after selection of the control population. In all cases, written informed consent was obtained from the guardians of the patients after explaining the detailed treatment protocol as per the patient information sheet. The study design and clinical procedures were performed in accordance with the Helsinki Declaration (revised in 2008) and were approved by the Ethics Committee of the School and Hospital of Stomatology, Fujian Medical University.

### PRF preparation

**Fig. 1:**
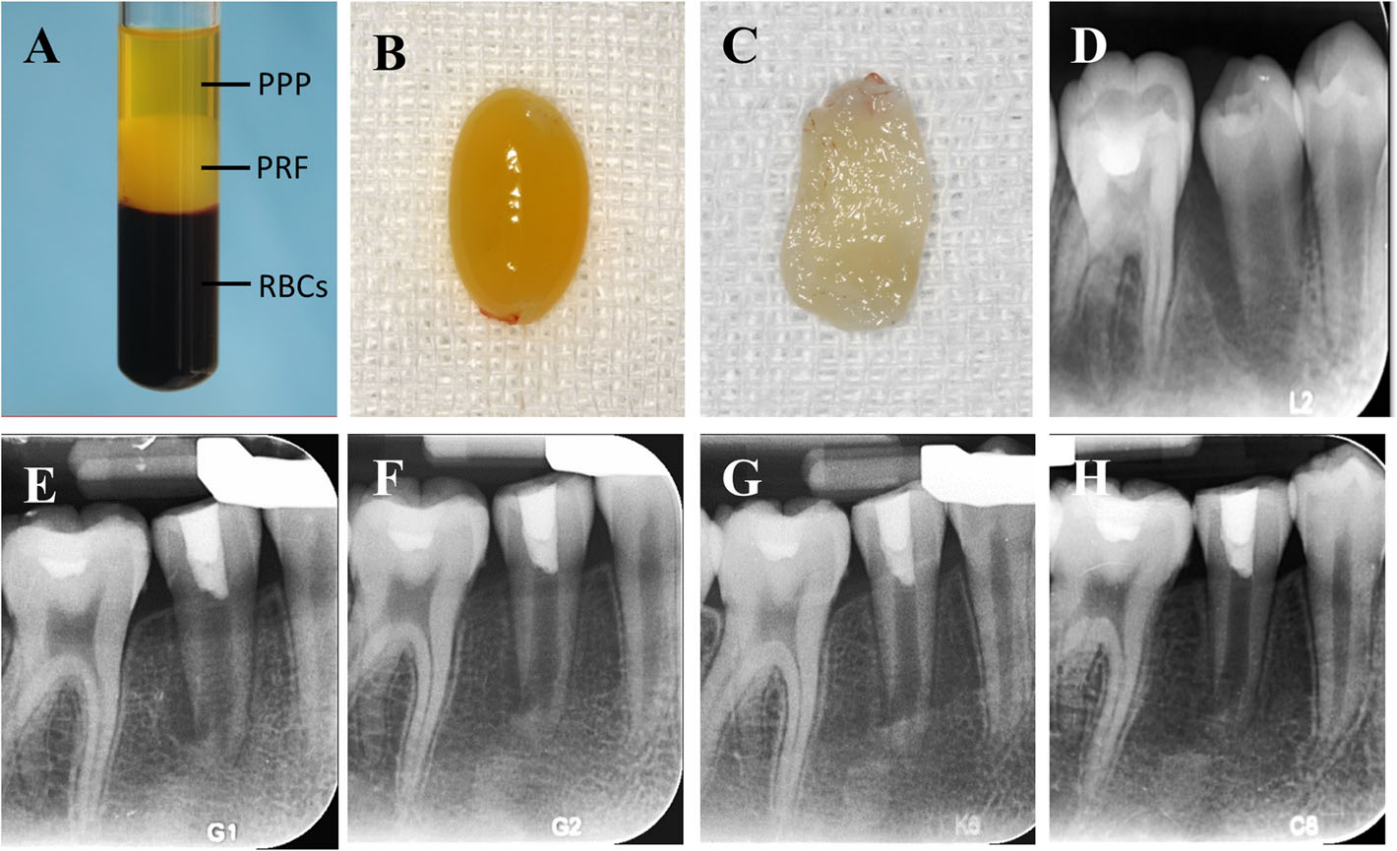
**a** Peripheral blood after centrifugation: red blood cells at the bottom, PRF in the middle, and platelet-poor plasma at the top. **b** PRF clot. **c** PRF membrane. **d** An periapical radiograph of #45 with apical periodontitis in a 12-year-old girl. The case was treated by RET+PRF in #45. **e** Three-month follow-up periapical radiograph of tooth #45. **f** Six-month follow-up radiograph. **g** Nine-month follow-up radiograph. **h** Twelve-month follow-up radiograph showing complete periapical radiolucency resolution, root apex closure, root elongation and root canal wall thickening

PRF was prepared as described by Choukroun et al. [33]. Immediately before surgery, 5 ml of whole blood was drawn into 10-ml test tubes without anticoagulant reagent and was centrifuged at 400 g for 10 min. After centrifugation, whole blood was divided into three layers: (1) the bottom red blood cell layer; (2) the middle PRF layer; and (3) the top serum layer. The PRF layer was separated using sterile scissors, and PRF clots were pressed into a membranous film with sterile dry gauze (Fig. 1a–c). The PRF membrane was then cut into approximately 3 × 3-mm^2^ pieces.

### Treatment procedure

The treatment procedure for tooth revascularization has been described in our previous study [5]. An access cavity was prepared under rubber dam isolation using a round diamond and an Endo-Z bur (Dentsply Maillefer, Tulsa, OK). The pulp chamber and root canal were gently irrigated with 20 mL of 1% NaOCl without mechanical instrumentation. The canal was then dried using sterile paper points. Subsequently, an inter-appointment medication of triple antibiotic paste comprising ciprofloxacin, metronidazole, and cefaclor (1:1:1) was placed into the apical portion of the canal and filled to just below the CEJ using a syringe under a microscope. The access cavity was temporarily restored with 3 mm of Cavit (ESPE, Seefeld, Germany) and 2 mm of glass ionomer (Fuji IX, GC, Tokyo, Japan).

Revascularization was performed 4 weeks later. The procedure that was used for conventional tooth revascularization was as follows: 2% lidocaine without adrenaline was infiltrated around the apex of the tooth. After reopening of the access, the antibiotic paste was gently flushed out of the canal with sterile normal saline. The irrigation was finalized with 10 mL of 17% EDTA solution, and the tooth was dried using sterile paper points. Under a surgical microscope (Carl Zeiss Meditac Inc., Dublin, CA), a sterile #35 K-file was introduced into the canal beyond the apical foramen using a push and pull motion to provoke bleeding from the periapical tissue. A sterile moist cotton pellet was placed 3 mm below the CEJ with gentle pressure for 15 min to form a BC in the root canal. The BC was directly covered by a layer of CollaPlug (Zimmer Dental, Carlsbad, CA). Then, 3 mm of ProRoot mineral trioxide aggregate (MTA) (Dentsply Tulsa Dental Specialties, Tulsa, OK) was placed over the CollaPlug. After a moist cotton pellet was placed over the MTA, the access cavity was sealed with Cavit.

Revascularization using PRF as scaffolding was performed as follows: local anaesthesia, access reopening, antibiotic paste removal and root canal irrigation were performed following the same procedure as that described previously. After final irrigation of the root canal with EDTA and drying using paper points, the PRF fragments were placed into the canal space using a Buchanan Hand Plugger Size #2 (Sybron Endo, Orange, CA) up to the CEJ. A 3-mm-thick layer of MTA was placed directly over the PRF, followed by a moist cotton pellet and Cavit. One week later, the Cavit was removed and replaced with a bonded resin restoration (Filtek Z350 XT: 3 M ESPE Dental Products, St Paul, MN).

### Postoperative examination and data collection

**Fig. 2:**
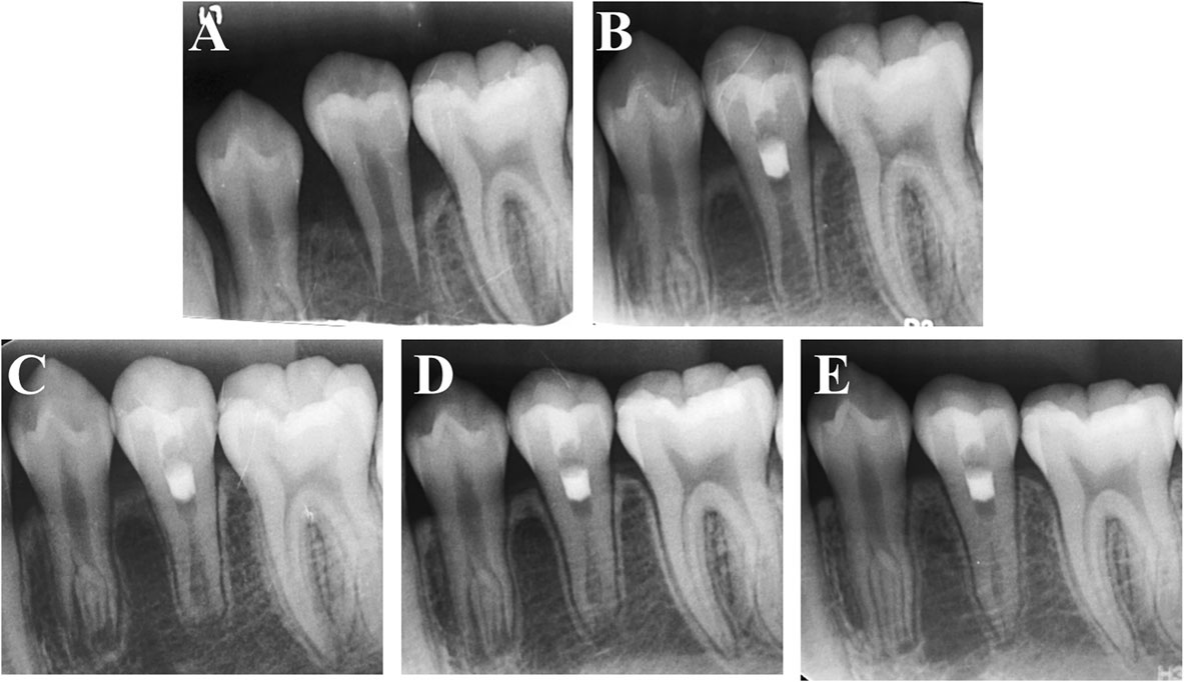
**a** An intraoral periapical radiograph of #35 with apical periodontitis in a 12-year-old girl. **b** Three-month follow-up periapical radiograph of tooth #35 after undergoing PRF-aided revascularization. **c** Six-month follow-up radiograph. **d** Nine-month follow-up radiograph. **e** Twelve-month follow-up radiograph showing complete resolution of the periapical radiolucency, thickening of the lateral dentinal walls, and closure of the apex

**Fig. 3:**
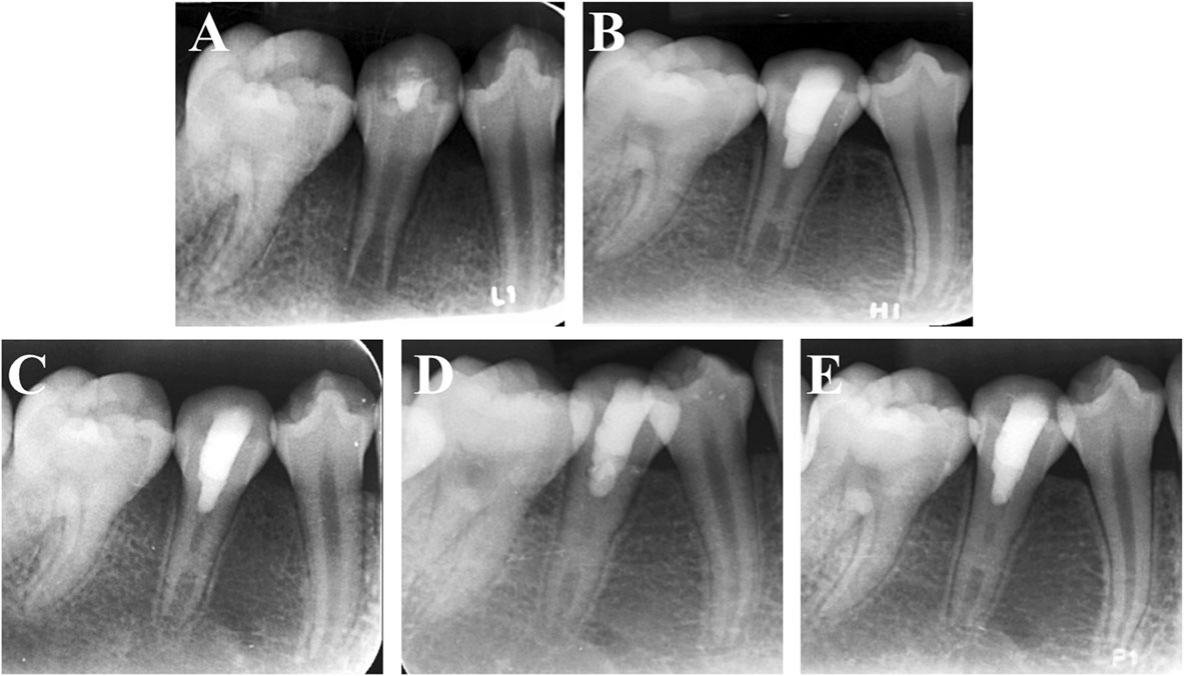
**a** A periapical radiograph of #45 with apical periodontitis in a 12-year-old girl. In #45, conventional RET was performed. **b** Three-month follow-up periapical radiograph. **c** Six-month follow-up radiograph. **d** Nine-month follow-up radiograph. **e** Twelve-month follow-up radiograph showing healing of the periapical lesion and root development

**Fig. 4:**
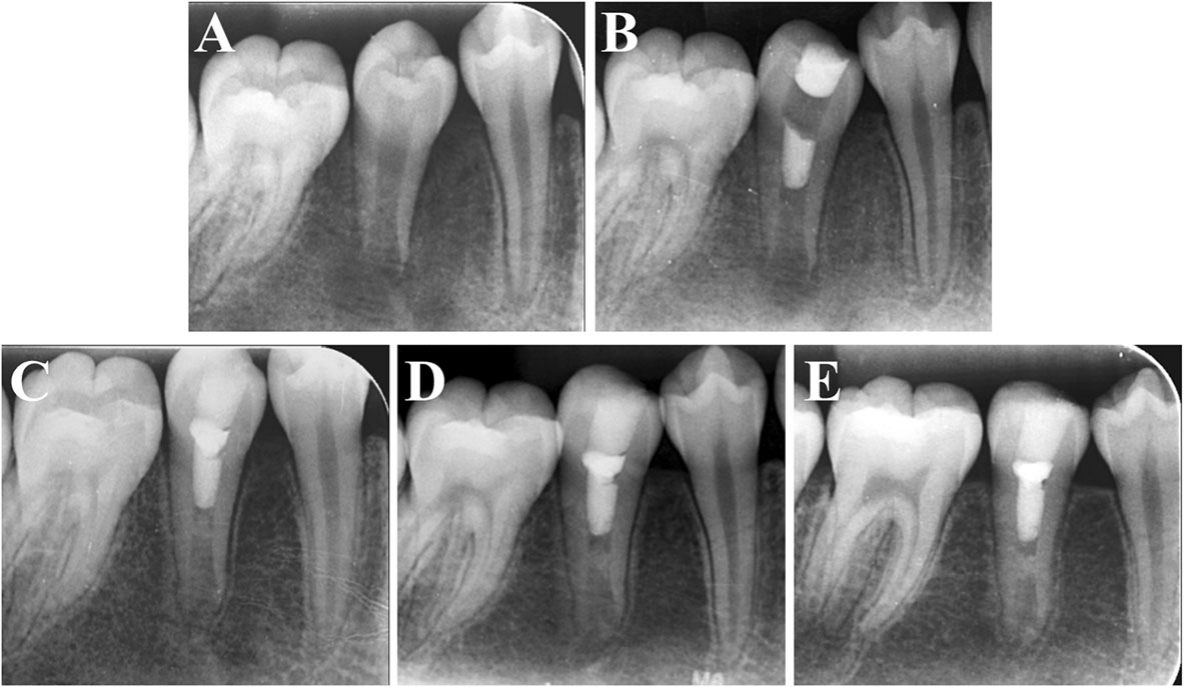
**a** An intraoral periapical radiograph of a 12-year-old girl showing a wide-open apex of #45 with thin lateral dentinal walls and apical radiolucency. **b** Three-month follow-up periapical radiograph of tooth #45 after undergoing conventional RET. **c** Six-month follow-up radiograph. **d** Nine-month follow-up radiograph. **e** Twelve-month follow-up radiograph showing apical radiolucency resolution, apex closure, root elongation and lateral dentinal wall thickening

Recall visits were scheduled at 3, 6, 9, and 12 months. At each appointment, clinical examination as well as a tooth sensibility test including an electronic pulp test (EPT) and a cold test were performed. Root development was monitored by periapical radiography taken with a paralleling technique using the same device (Fig. 1d–h, Figs. 2, 3 and 4).

### Statistical analyses

Statistical analyses was performed using SPSS (Statistics software v 19.0; IBV Corp, Armonk, NY). The results were compared between groups using the Chi-Square test. *P* < 0.05 was considered statistically significant.

## Results

The results obtained are summarized in Tables 1 and 2. A total of 5 males and 5 females were included in this study. Mean patient ages for the BC and PRF groups were 11.6 (10–12) and 11.4 (9–14) yr., respectively. Compared to the BC group, more males were included in the PRF cohort, but the difference was not significant (60% vs 40%, *P* > 0.05). In each group, one incisor and 4 premolars were treated (Table 1). Postoperatively, resolution of periapical radiolucency was observed in all treated cases (100%). Root elongation, lateral dentinal wall thickening and apex closure were detected in most cases (80% in both groups). Clinical sign and symptom resolution was observed in all patients (100%). There was no significant difference between the groups in terms of root development, periapical healing and clinical sign resolution (*P* > 0.05) (Table 2).

**Table 1:** Clinical and radiographic findings of teeth receiving RET with BC or PRF as scaffold at 1-year Follow-up

Procedure performed	Preoperative variables				Postoperative variables at 1-year follow-up					
					Radiographic findings				Sign and symptom	Sensitivity test
	Tooth# (age/sex)	Aetiology	Pulp/periapical conditions	Foramina development (Nolla’s stage)	Periapical healing	Apex closure	Root elongation	Dentinal wall thickening	Resolution	EPT/Cold test (time)
RET with a BC (average age = 11.6 y)	45 (10 y/F)	DE	AP	8	Yes	Yes	Yes	Yes	Yes	+(6 m)
	45 (12 y/M)	DE	AP	8	Yes	Yes	Yes	Yes	Yes	–
	45 (12 y/F)	DE	AP	8	Yes	Yes	Yes	Yes	Yes	–
	45 (12 y/F)	DE	AP	8	Yes	Yes	Yes	Yes	Yes	–
	11 (12 y/M)	Tooth fracture	AP	8	Yes	No	No	No	Yes	–
RET with PRF (average age = 11.4 y)	35 (10 y/M)	DE	AP	8	Yes	Yes	Yes	Yes	Yes	–
	45 (12 y/F)	DE	AP	8	Yes	Yes	Yes	Yes	Yes	+ (6 m)
	35 (12 y/M)	DE	AP	8	Yes	Yes	Yes	Yes	Yes	+ (9 m)
	45 (14 y/F)	DE	AP	8	Yes	No	No	No	Yes	–
	21 (9 y/M)	Tooth fracture	AP	8	Yes	Yes	Yes	Yes	Yes	+ (9 m)

*RET* regenerative endodontic treatment, *BC* blood clot, *PRF* platelet-rich fibrin, *F* female, *M* male, *DE* dens evaginatus, *AP* apical periodontitis; +, positive; −, negative

**Table 2:** Comparative evaluation of tooth revascularization with BC and PRF in terms of clinical and radiographic findings at 1-year follow-up (* *P* < 0.05)

	Radiographic findings at 1-year follow-up				Clinical sign and symptom resolution
Procedure performed	Periapical healing	Apical closure	Root lengthening	Dentinal wall thickening	
RET with a BC	5 (100%)	4 (80%)	4 (80%)	4 (80%)	5 (100%)
RET with PRF	5 (100%)	4 (80%)	4 (80%)	4 (80%)	5 (100%)

## Discussion

The data obtained in the present study showed that tooth revascularization/revitalization using PRF as a scaffold achieved comparable results to the technique of provoking periapical bleeding in terms of periapical lesion healing, continued root formation and clinical sign and symptom resolution. To the best of our knowledge, this is the first retrospective controlled cohort study to compare the efficacy of PRF and BC as a scaffold in RET.

The present study showed no significant difference between PRF and BC groups in terms of periapical lesion healing, root development and clinical sign and symptom resolution. Because we did not provoke periapical bleeding before PRF placement in this study, periapical radiolucency resolution and root development were caused by the presence of the PRF scaffold. Our result is consistent with most previous studies [14, 34, 35] in which PRP served as the only bioscaffold in the treatment protocols. Although another study [24] revealed a remarkable enhancement of periapical healing, apical closure, and dentinal wall thickening in their PRP group, PRP was applied after provoking periapical bleeding in the treatment protocol. The superior performance of the PRP group in that study might be due to the synergistic effects of a BC and PRP in root development. On one hand, the concentrations of CD73 and CD105 mesenchymal stem cells in blood samples taken from immature teeth was up to 600-fold greater than that in circulating blood [36]. The stem cell population in periapically-induced BC should be significantly higher than that in PRF, which is prepared from peripheral blood. On the other hand, compared to a BC, PRF contains a much higher concentration of platelets [37], which might continuously release various growth factors, thereby contributing to tissue regeneration. In a pilot study, Narang et al. also compared the efficacy of PRF scaffold with that of a BC in RET. It was found that PRF achieved comparable effects in apical closure, dentinal wall thickening and even better results in periapical healing and root lengthening than a BC did [31]. As the patients’ ages in their study differed from those in ours, the stem cell concentration in the BC might not be at the same levels. This could possibly affect the results of the treatment. Anyway, our results along with previous studies showed that the effect of PRF as a scaffold in RET was at least comparable, if not superior to that of a BC from the periapical region.

Apart from periapical radiolucency resolution and root development, tooth sensibility recovery has also been reported in many previous studies [1, 3, 13, 38–40]. In these studies, positive responses of teeth to cold and EPT were detected from 5 1/2 months to 2 years postoperatively. In our study, positive responses to the tooth sensibility test were observed between 6 and 9 months after RET. However, because the follow-up period of the present study was only 12 months, tooth sensibility recovery after 1 year was not recorded. Therefore, at this point, we are cautious in drawing conclusions regarding whether PRF scaffold achieves better functional outcomes in terms of pulp sensitivity than a BC. Long-term observations are needed in future studies.

Due to the lack of specimens of human teeth showing revascularization/revitalization, the underlying histological basis for the presence or absence of responses in the tooth sensitivity test remains unclear. Hargreaves et al. proposed that positive responses to pulp sensitivity tests after tooth revascularization/revitalization indicate the occupation of previously vacant space by innervated tissue [41]. Johns and Vidyanath suggested that thick layers of MTA (3–4 mm) and glass ionomer cement (2 mm) might lead to negative responses to vitality testing [42]. Most recently, we reported a histological study regarding the nature of newly formed tissues after tooth revascularization [5]. In that case, an immature mandibular premolar with apical periodontitis was treated by revascularization/revitalization. Successful treatment results were observed including periapical radiolucency resolution, root development and tooth sensitivity recovery. Ten months later, the tooth was extracted for orthodontic reasons and processed for histological observation. In the canal space, neurons and nerve fibres were observed histologically and were confirmed by immunohistochemical examination. This finding demonstrated the possibility of nerve regeneration after RET, which may play a key role in the recovery of tooth sensitivity.

It has been documented that the mental foramen is located close to the mandibular premolars. In a radiographic study conducted by Fishel D et al. [17], the mental foramina were located at the apices of the first or second premolars in 15.4% or 13.9% of patients, respectively. In another study that aimed to determine the position of the mental foramen in relation to the apex of the second premolar [15], Phillips JL observed that the apex of the second premolar was located anywhere between 2.7 mm mesial, 3.8 mm distal, 3.5 mm above or 3.4 mm below the mental foramen. The author summarized that the apex of the second premolar was located at an average distance of 2.18 mm mesially and 2.4 mm superiorly from the mental foramen. The proximity of the apex of the second premolar and the mental foramen was also confirmed in a cadaver study by Denio D et al. [16]; their study demonstrated that each mental foramen was between 0 mm and 4.7 mm from the respective apex of the second premolar. Obviously, manipulation using needles or files to provoke bleeding beyond the apical foramen of premolars carries the risk of nerve injury. Therefore, it is rational and imperative to find a safe and efficient alternative to periapical bleeding in RET under such situations.

Despite the small number of cases observed, our study provides useful information on the clinical outcome of using PRF in scaffold-enhanced periapical lesion rehabilitation and root development in RET. These data demonstrated the feasibility of using PRF as an alternative scaffold when treating mandibular premolars or when provoking apical bleeding proves difficult. The major disadvantages of using PRF include the need to draw blood in young patients and the need for specialised equipment. However, considering the risks of nerve injury, the advantages of PRF application apparently outweigh its disadvantages in certain cases. Nevertheless, more randomized prospective controlled studies are needed to confirm the reliability of PRF for use as a bioscaffold to aid in developing future guideline recommendations for tooth revascularization/revitalization.

## Conclusions

In sum, within the limits of this study, PRF achieved comparable outcomes to BC in terms of clinical sign and symptom resolution, periapical lesion healing and root maturation in RET.

## Abbreviations

BC: Blood clot
CEJ: Cemental enamel junction
EPT: Electronic pulp test
IAN: Inferior alveolar nerve
MTA: Mineral trioxide aggregate
NaOCl: Sodium hypochlorite
PDGF: Platelet-derived growth factor
PRF: Platelet-rich fibrin
RET: Regenerative endodontic treatment
TGF: Transforming growth factor
VEGF: Vascular endothelial growth factor
